# Do the Hydatid Cysts Have Unusual Localization and Dissemination Ways in the Chest Cavity?

**DOI:** 10.1155/2016/7092494

**Published:** 2016-03-29

**Authors:** Yucel Akkas, Tevfik Kaplan, Neslihan Gulay Peri, Bulent Kocer

**Affiliations:** ^1^Department of Thoracic Surgery, Ankara Numune Training and Research Hospital, Ankara, Turkey; ^2^Department of Thoracic Surgery, Ufuk University Faculty of Medicine, Ankara, Turkey

## Abstract

We wanted to report our two cases of intrathoracic extrapulmonary hydatid cyst in pleural cavity due to its rarity. Our first case is a 24-year-old male patient who was admitted with a cystic mass lesion consistent with hydatid cyst which was incidentally detected in inferior lobe of the right lung neighboring to thoracic wall and diaphragm. Our second case is a 32-year-old male patient who was admitted with chest pain and a cystic lesion in apex of the right hemithorax and intercostal field in basal after he had been medically treated due to hydatid cyst of the dome of the liver for two years. The cysts were removed with thoracotomy. Extrapulmonary intrathoracic hydatid cysts were evaluated with regard to invasion ways and treatment indications and methods.

## 1. Introduction

Hydatid cyst is a parasitic disease which is common in endemic regions and seen most frequently in the liver followed by the lungs. Intrathoracic extrapulmonary cysts are rarely seen. These cysts are localized in fissure, pleural cavity, chest wall, mediastinum, myocardium, and diaphragm, most commonly in pleural cavity [1, 2].

We wanted to report our two hydatid cysts located in pleural cavity due to its rarity.

## 2. Case 1

**Figure 1:**
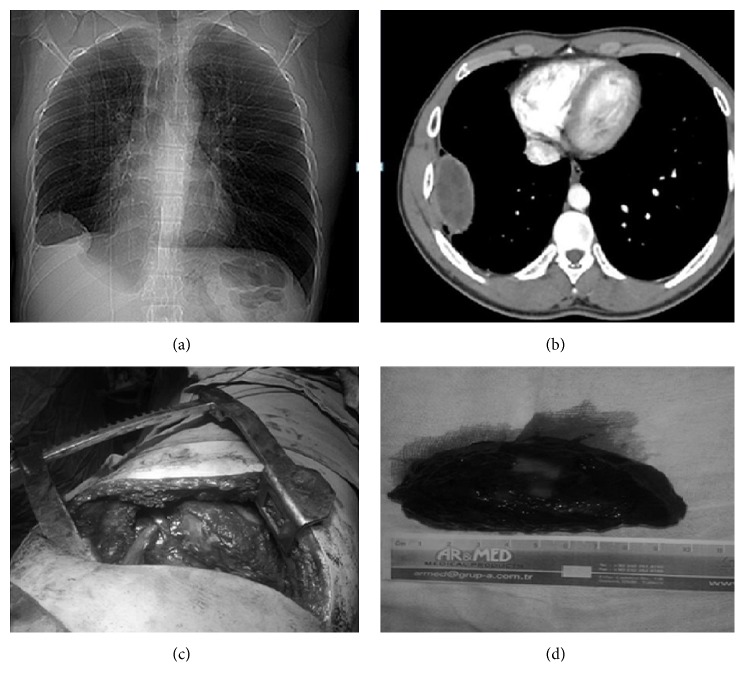
Chest X-ray image of the extrapulmonary intrathoracic hydatid cyst in the first case (a). Computed tomography image of the extrapulmonary intrathoracic hydatid cyst in the first case (b). Operation images of the first case (c). Image of the totally resected hydatid cyst (d).

A 24-year-old male patient was admitted to our hospital due to a mass lesion with regular contours located in costodiaphragmatic sinus in the right inferior zone which was detected in posteroanterior chest X-ray during his routine control (Figure 1(a)). The patient had no complaints. Computed tomography of thorax revealed a mass lesion measuring 6 × 10 × 3 cm, neighboring to chest wall and diaphragm in inferior lobe of the right lung, consistent with hydatid cyst (Figure 1(b)). Laboratory data were normal. Right lateral thoracotomy was performed. On exploration, a firm, extraparenchymal cystic lesion measuring 10 × 6 cm, beginning from the inferior of the 6th rib, adhered to pleura was totally resected. The cyst had no association with parenchyma (Figures 1(c) and 1(d)). The patient was discharged on postoperative day 7. Pathology result was reported as hydatid cyst. 15–20 mg/kg daily albendazole was administered to him for 2 postoperative months. No postoperative complications or recurrence occurred in the patient who is on postoperative month 18.

## 3. Case 2

**Figure 2:**
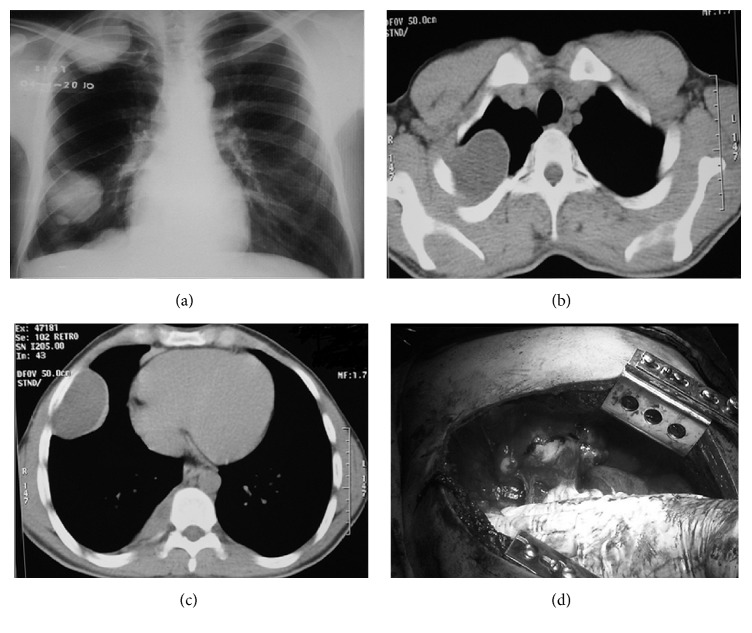
Chest X-ray image of the extrapulmonary intrathoracic hydatid cyst in the second case (a). Computed tomography image of the hydatid cyst in apex of the right hemithorax (b). Computed tomography image of the hydatid cyst in intercostal field (c). Operation image of the second case (d).

The 32-year-old male patient had received medical therapy due to hepatic hydatid cyst 2 years ago. The patient who had chest pain 2 years after the first diagnosis was detected to have a cystic lesion measuring 6 × 6 × 10 cm in apex of the right hemithorax and 4 × 4 × 10 cm in intercostal space basal on his control chest X-ray and computed tomography of thorax (Figures 2(a), 2(b), and 2(c)). The extraparenchymal firm cystic lesions were totally resected with right thoracotomy (Figure 2(d)). The patient was discharged on postoperative day 7. Pathology result was reported as hydatid cyst. 15–20 mg/kg daily albendazole was administered to the patient for 2 months postoperatively. He is on 3rd postoperative year and he did not develop any problems or recurrence.

## 4. Discussion

Hydatid cyst is a parasitic disease caused by* Echinococcus granulosus* which is seen in developing countries like Turkey. While hydatid cysts can be seen in all organs, it is seen in extrapulmonary intrathoracic region in the ratio of 7,4% [3]. Among them, pleural cavity is the most common localization [1].

While hydatid cyst can be asymptomatic, it can also cause various symptoms. Extrapulmonary intrathoracic hydatid cysts may lead to symptoms due to compression to neighboring tissues. Gürsoy et al. [4] reported chest pain and dyspnea due to pulmonary pressure in the ratio of 70%. While our first case was asymptomatic due to the mass lesion not being excessively big, the second case had chest pain which is the most common symptom.

Differential diagnosis of extrapulmonary intrathoracic hydatid cysts includes diaphragmatic, dermoid, neurenteric, or neurenteric duplication cysts or masses. Making a differential diagnosis is difficult. Imaging methods like posteroanterior, lateral chest X-ray, ultrasonography, computed tomography of thorax, and magnetic resonance imaging may be used for differential diagnosis [2, 5]. We used computed tomography of thorax for differential diagnosis and detection of the localization after we had detected the cysts with posteroanterior chest X-ray which was obtained incidentally in the first case and due to being symptomatic in the second case.

Contrary to the standard hydatid cyst invasion, Isitmangil et al. [6] reported that hydatid cysts of the dome of the liver proceed to diaphragm and scolexes enter thorax via diaphragmatic lymphatics and invaded through parasternal lymph nodes anteriorly and through intercostal lymph nodes posteriorly. Atoini et al. [7] suggested that there is a different route of dissemination from the standard hydatid cyst invasion. Foroulis et al. [5] reported that four cases were intrathoracic extrapulmonary hydatid cysts. Three of these four cases were cases of intrathoracic extrapulmonary hydatid cysts in patients with multiorgan involvement but one of the patients was with pleural site as the only location of the disease. We consider that invasion occurred from liver to thorax through these ways, particularly in case 2.

Intrathoracic extrapulmonary hydatid cysts are treated with resection of the cyst. Özyurtkan and Balci [8] suggested that total excision was performed for the intrathoracic extrapulmonary hydatid cysts. Good results of surgical resection in patients with chest wall hydatid cysts were reported in the literature [5, 9]. In spite of this, some authors suggested the percutaneous treatment of hydatid cysts and aspiration and alcohol injection under sonographic guidance [10, 11]. These may be considered for inoperable cases or for patients who refuse surgery because of their complications [12]. In our first case, we totally resected the cysts in order not to enlarge and cause compression on neighboring tissues despite being asymptomatic and we totally resected the cyst in the second case as it was symptomatic [1].

We administered albendazole treatment for two months postoperatively in order to prevent secondary recurrence in both cases [2].

## 5. Conclusion

Hydatid cyst may rarely be localized in thorax out of the lung parenchyma. Extrapulmonary intrathoracic cysts may also develop through invasion of the hydatid cysts of the dome of the liver through diaphragmatic lymphatics besides normal invasion and they should be immediately resected surgically when detected as they may cause compression on intrathoracic organs when they enlarge.
